# Supplementary material for: Microscale Lateral Perovskite Light Emitting Diode Realized by Self-Doping Phenomenon
Source: Sensors (Basel). 2024 Jul 10;24(14):4454. doi: 10.3390/s24144454 (PMC11280541; doi:10.3390/s24144454)
Supplement: Supplementary file 1 [file sensors-24-04454-s001.zip › sensors-3037399-supplementary.pdf]

sensors

MDPI

Supporting Information

Microscale Lateral Perovskite Light Emitting Diode Realized by Self-doping Phenomenon

Wenzhe Gao[1,†], He Huang[2,†], Chenming Wang[3], Yongzhe Zhang[1], Zilong Zheng[2,*], Jinpeng Li[3,*], Xiaoqing Chen[1,*]

As shown in Figure S1, AC pulse voltage (the period is 100 ms, and low-level input voltage is 0 V, duty cycle is 50%) is applied to the devices of different channels. For devices with 2 μm and 3 μm channels, the high-level input voltage of 5V emits better excitation light, and for devices with 5 μm channels, a high input voltage is 10V. When the appropriate polarization voltage is applied, the devices with 2 μm and 3 μm channels generally show longer (about 3 s) illumination duration and larger illumination area, while the devices with 5 μm channels show shorter (about 1 s). Overall, the 3 μm channel device has better repeatability than 2 μm. Since the prime contribution of this paper is the microscale dimension of the perovskite light emitting device, we chose to use the 2 μm device in our manuscript.

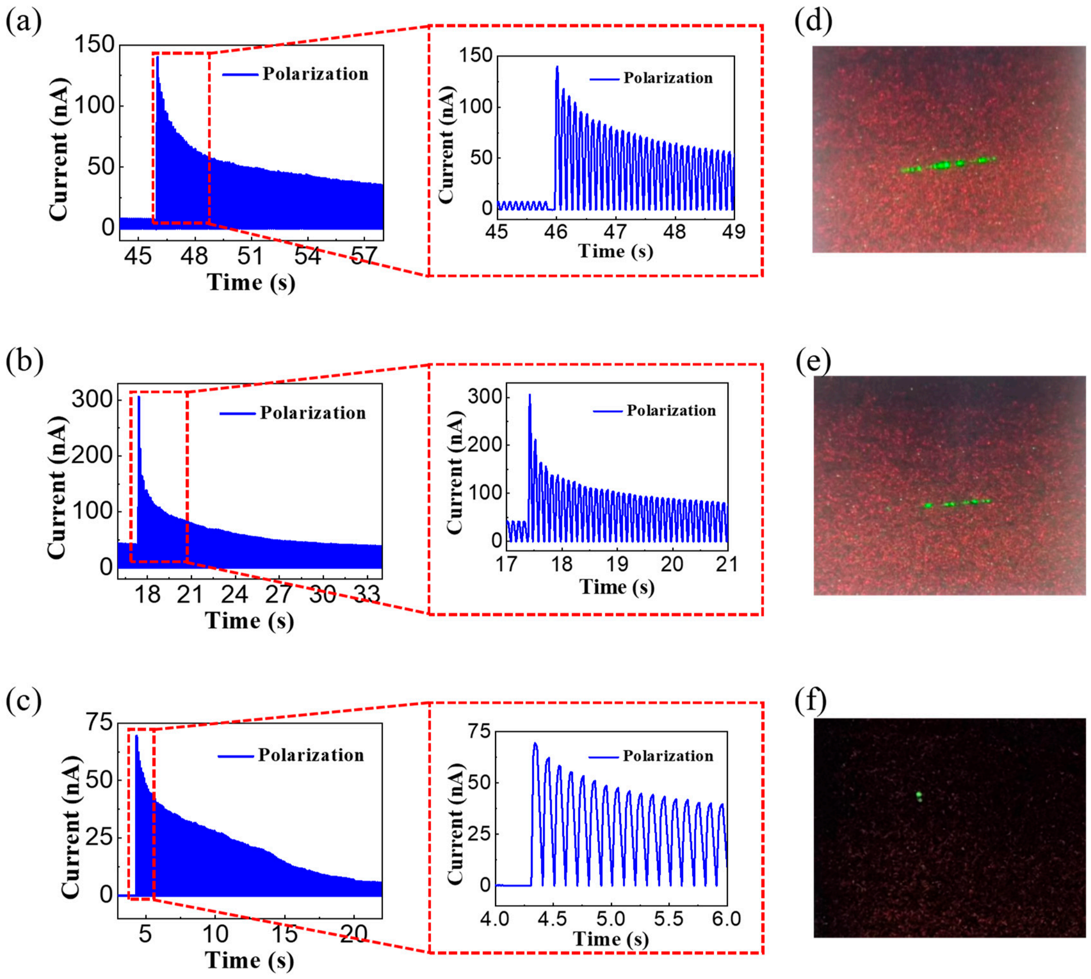

Figure S1. The I-t output curves of the devices with different channel widths: (a) 2 μm, (b) 3 μm, (c) 5 μm. Luminescence images under optical microscope of the devices with different channel widths: (d) 2 μm, (e) 3 μm, (f) 5 μm.
